# Flash Electroretinography as a Measure of Retinal Function in Myopia and Hyperopia: A Systematic Review

**DOI:** 10.3390/vision7010015

**Published:** 2023-02-27

**Authors:** Sania Zahra, Melanie J. Murphy, Sheila G. Crewther, Nina Riddell

**Affiliations:** 1 Department of Psychology, Counselling and Therapy, La Trobe University, Melbourne 3083, Australia; 2 Centre for Human Psychopharmacology, Swinburne University of Technology, Melbourne 3122, Australia

**Keywords:** electroretinogram, ERG, myopia, hyperopia, refractive error

## Abstract

Refractive errors (myopia and hyperopia) are the most common visual disorders and are severe risk factors for secondary ocular pathologies. The development of refractive errors has been shown to be associated with changes in ocular axial length, suggested to be induced by outer retinal elements. Thus, the present study systematically reviewed the literature examining retinal function as assessed using global flash electroretinograms (gfERGs) in human clinical refractive error populations. Electronic database searching via Medline, PubMed, Web of Science, Embase, Psych INFO, and CINAHL retrieved 981 unique records (last searched on the 29 May 2022). Single case studies, samples with ocular comorbidities, drug trials, and reviews were excluded. Demographic characteristics, refractive state, gfERG protocol details, and waveform characteristics were extracted for the eight studies that met the inclusion criteria for the review and were judged to have acceptable risk of bias using the OHAT tool (total *N* = 552 participants; age 7 to 50). Study synthesis suggests that myopia in humans involves attenuation of gfERG photoreceptor (a-wave) and bipolar cell (b-wave) function, consistent with the animal literature. Meaningful interpretation of the overall findings for hyperopia was limited by inconsistent reporting, highlighting the need for future studies to report key aspects of gfERG research design and outcomes more consistently for myopic and hyperopic refractive errors.

## 1. Introduction

Refractive errors (myopia and hyperopia) are the most common visual disorders [1] and pose a growing socioeconomic and public health problem. They occur when the length of the eye and refractive power of optical components prevents accurate focusing of light on the neural retina. A myopic (short-sighted) eye is excessively large, while hyperopia occurs when the eye is too small. Myopia affects >1.5 billion people globally, with prevalence estimated to increase dramatically to ~50% of the global population by 2050, a figure purported to be driven by increased educational and near-work demands [2,3,4]. High myopia of more than six diopters (D) affects a significant proportion of myopes, placing them at very high risk of developing severe vision threatening secondary pathologies such as retinal detachment, glaucoma, and choroidal neovascularization later in life [3,4,5]. Low myopes are also at risk of pathological complications such as maculopathy [6], further emphasizing the clinical significance of this condition and the need to better understand its etiology. Thus, to allow exploration of the site of the retinal elements contributing to myopia development, this paper aimed to systematically review the current literature associated with electrophysiological measurement of functioning of all cell types in the retina using global flash electroretinograms (gfERGs) rather than the more common pattern ERG that primarily provides information about the functioning of neurons in the inner retina ([7]) of clinically defined myopic and hyperopic humans. 

### 1.1. Mechanisms Controlling Eye Growth and the Development of Refractive Errors 

Although myopia and excessive eye growth are associated with heritable genetic contributions, the post-1975 myopia epidemic is considered to be environmentally driven [8]. Environmental factors such as education and limited time outdoors have been associated with dramatic increases in myopia prevalence in children and young adults in East Asia (for example, from 26% in young adults in Singapore in the 1970s to 83% around 2000) [9,10,11].

Although refractive myopia can be optically corrected with spectacles, contact lenses, and laser surgery, there is no treatment known to effectively inhibit the progressive ocular growth and changes in retinal morphology, choroidal thickness, and blood flow that increases secondary pathology risk once it is initiated [12]. Lifestyle changes and increased hours outside can delay the onset of myopia in children [13], but these approaches do not prevent myopia, highlighting the need for further research into etiology and strategic management. Animal studies have demonstrated that removing communication between the retina in the eye and higher visual centers in the brain via ciliary nerve or optic nerve section does not prevent ocular growth in response to visual manipulations designed to produce myopic and hyperopic refractive errors [14,15]. This suggests that the mechanisms regulating ocular growth are local to the light-sensing retina of the eye.

Multiple theories exist regarding the biological mechanisms and cell types within the retina that are directly involved in regulating ocular growth, with many postulating that the function of the outer retina and ON/OFF bipolar cell pathways may be particularly important [16,17]. The retina has a laminar structure, with the outer retina composed of rod and cone photoreceptors that transduce light into a neural signal that is transmitted to bipolar and ganglion cells in the inner retina. Horizontal and amacrine cells in the inner retina act to further modulate the ascending signal. Different theories have proposed a role for the photoreceptor/RPE interface [18,19], ON and OFF bipolar cells [20,21], amacrine cells [22], and various signaling molecules and pathways throughout the inner and outer retina [16] in controlling eye growth and inducing refractive errors. In animal models, neurotoxic agents that disrupt amacrine and ganglion cell functioning alone have little effect on the growth of the vitreous chamber [23,24,25,26], while those that affect bipolar or photoreceptor function do alter the rate of postnatal vitreous enlargement and visually induced ocular growth [21,23,24,27,28], suggesting that functional changes at the photoreceptor/RPE/bipolar cell interface may be central to myopia etiology. Recent studies of genetic variants associated with a greater risk of myopia in humans have also implicated photoreceptor and bipolar cell function [17,29], further suggesting that outer retinal function is central to the pathophysiology of the disorder.

### 1.2. Electrophysiology as a Technique for Understanding the Role of Retinal Cells in Human Refractive Errors

Electroretinography can be used to non-invasively assess phototransduction and retinal processing of light at the cellular level in humans and animals [17]. The Electroretinogram (ERG) is an established electrophysiological diagnostic technique that is widely used in clinical and laboratory settings [30,31]. ERGs utilize external electrodes to measure the electrical activity of the retina following a light stimulus, such as a bright flash. Variations in the stimulus and recording setup allow multiple types of ERGs to be recorded, including Pattern (PERG), multifocal (mfERG), and global flash (gfERG), each differing in the specific information that they provide about retinal function [30,31]. In contrast to other ERG types (such as inner retinally focused PERGs), the waveform recorded in the gfERG predominantly reflects the global function of outer retinal photoreceptors, bipolar, RPE and Muller cells that have been theorized to play a key role in driving ocular growth changes and are also associated with the later development of secondary pathologies in myopia [32].

### 1.3. Using the gfERG to Functionally Dissect Retinal Activity 

**Figure 1:**
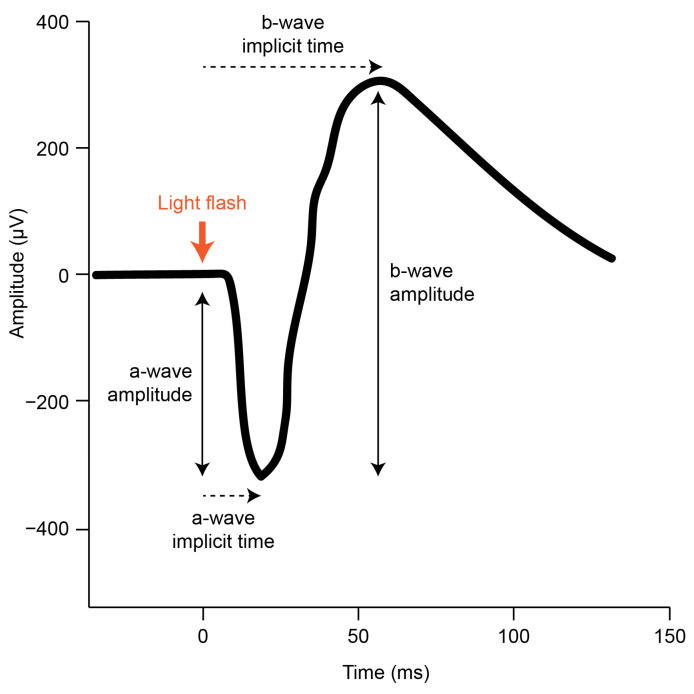
A typical scotopic gfERG initiated by a brief light flash (adapted from the ISCEV standard [31]). The onset of the light flash is indicated (thick orange arrow) alongside the a-wave and b-wave amplitude and implicit time components that can be measured in the resulting retinal response.

The gfERG is a measure of the mass response of the retina, elicited by a brief flash of light, that has been used to assess generalized retinal function in a broad range of ophthalmic conditions including refractive errors [33]. The gfERG response can be produced under various conditions including dark adaptation (to isolate the scotopic rod response) and light-adaptation (to isolate the photopic cone response) [31]. The gfERG waveform (Figure 1) reflects a series of current loops that redistribute ions within the extracellular spaces of the retina after the onset of the light flash. These currents result primarily from changes to photoreceptor and bipolar cell polarity and subsequent interactions with Muller glial and RPE cells. Clinical gfERGs typically capture two major components in the recorded waveform; the a-wave primarily reflects the activity of the rod and cone photoreceptors, and the b-wave primarily reflects the activity of the bipolar cells to light onset [33,34]. Further analysis can uncover small rhythmic wavelets during the ascending phase of the b-wave called oscillatory potentials, which primarily reflect inhibitory feedback by inner retinal amacrine cells [35]. The function of each cell type can be inferred by measuring the size of each wave (amplitude) and the difference in time between onset of the response and maximum response reached (implicit time). Consistent with the animal model pharmacological research outlined above, attenuation of the gfERG a-wave and b-wave amplitude has been demonstrated in studies of optically and pharmacologically induced myopia in chicks [36,37,38,39]. However, although gfERG has been used extensively in clinical settings to examine human myopia e.g., [40,41,42,43,44]; the findings have been mixed, and current knowledge has not been systematically reviewed.

### 1.4. Rationale and Aim of the Current Systematic Review

Although it is well accepted that the excessive ocular growth observed in animal models of myopia is locally controlled within the retina, the evidence and theories regarding the particular cell types involved in the control of ocular growth and the progression to secondary pathology in humans and animal models are still under debate [16,17]. Furthermore, to develop effective treatments and to facilitate the identification of those at higher risk of progressive myopic eye growth and later development of sight-threatening pathology, a better understanding of cellular contributions to myopia is necessary. As the gfERG measures the function of key cell types theorized to be involved in the onset and progression of myopia, synthesizing the evidence for gfERG changes in refractive error may provide insight for future research regarding the functional state of the retina over the course of the condition and the efficacy of interventions in preserving retinal integrity. Thus, the present paper aimed to systematically review the gfERG literature assessing retinal cell function in myopic and hyperopic humans. In accordance with PRISMA guidelines, our literature search and study selection strategies will be described, followed by our data extraction and risk of bias procedures. Following consideration of the study data extracted, results will examine data pertaining to gfERG waveform characteristics observed in refractive error in general and then for myopia and hyperopia, respectively. The discussion will examine these findings in context with the previous literature.

## 2. Methods

### 2.1. Literature Search

This review followed the PRISMA guidelines for systematic reviews [45]. PubMed, MEDLINE, Web of science, Embase, CINAHL, and PsycINFO databases were searched to identify all potentially eligible studies. This combination of databases was chosen for the coverage of concepts relevant to this review, and because it has been shown to retrieve more than 95% of all possible relevant references across a large selection of systematic reviews [46]. Google Scholar was not included due lack of specificity, accessibility, and search accuracy [47]. The final database search was conducted on 29 May 2022. Reference list searching of included studies was also conducted. This review was not registered prior to completion. Table 1 outlines the search strategy and provides example results from PubMed.

### 2.2. Study Selection 

Studies comparing gfERG measures across different refractive states (e.g., myopia, hyperopia, high myopia, emmetropia) in human subjects were included in the review. Intervention studies (e.g., drug studies) were only included if baseline data were available. Studies were excluded if they investigated refractive error as a peripheral measure secondary to other conditions that are expected to be associated with functional deficits (e.g., congenital stationary night blindness and single gene mutations associated with multifaceted phenotypes). Single-case studies and studies of non-human animals were also excluded from the review.

The screening process was performed using Covidence Systematic Review Data Management Software. The title and abstract screening were performed independently by two reviewers (SZ and NR), where each reviewer decided either to reject or accept each record based on the inclusion and exclusion criteria outlined above. Conflicts were resolved through discussion with a third reviewer (MM). The same process was applied to full-text screening to identify relevant studies for data extraction. Decisions on whether to reject a study were based on the seven-step hierarchy presented in Table 2.

### 2.3. Data Extraction 

A data extraction table was constructed in Microsoft Excel. Data were extracted independently by two reviewers (SZ and NR). Table 3 lists the types of information extracted from each included study. Due to inconsistency between studies in the reporting of results, both quantitative and qualitative data were extracted.

### 2.4. Risk of Bias Assessment 

Quality assessment of each included study was conducted using the Office of Health Assessment and Translation (OHAT) risk of bias tool for human and animal studies [48]. The OHAT is recommended by the National Health and Medical Research Council of Australia as a practical and flexible best practice tool for risk of bias assessment [49,50,51,52]. Questions 1, 2, and 5 from the tool (Table 4) were excluded from the present assessment as they were only applicable to either human-control trails or experimental animal studies. Question 11 provides the option for additional questions about other potential threats to internal validity to be added on a project-specific basis and hence was not used in the current review. The risk of bias assessment was independently completed by SZ, and where required all authors were consulted regarding judgements.

## 3. Results

### 3.1. Study Selection

**Figure 2:**
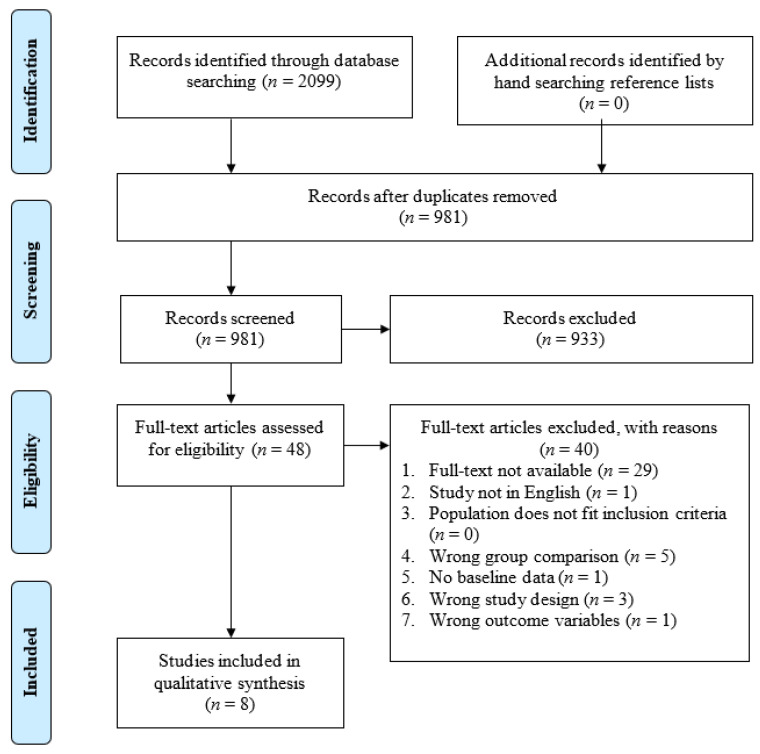
PRISMA flow diagram of the study selection process.

As seen in Figure 2, electronic database searching and hand searching of reference lists identified 981 unique records (*n* = 1118 duplicates). Of the 981 records that entered title and abstract screening, 933 studies were deemed irrelevant based on the inclusion criteria. Full-text screening of the remaining 48 records identified 40 studies that did not meet inclusion criteria and were excluded. Among the excluded studies, 29 did not have full-text available (e.g., primarily conference abstracts); five had the wrong group comparison; three had the wrong study design; one was not in English; one had no baseline data; and one had the wrong outcome variable. Eight studies that met the inclusion were included in the systematic review.

### 3.2. Risk of Bias Assessment 

Overall risk of bias using the OHAT tool was deemed to be either below the critical level or minimal across the studies except for two studies which demonstrated a definitely high risk of bias on question 7 “Were outcome data complete without attrition or exclusion from analysis?” (Table 5). Data from six participants for one study who were part of baseline data for comparison could not be obtained [50]. The other study did not obtain data from some participants for certain components of the ERG [41]. All included studies displayed a “probably high” risk of bias for the question 6 “Were the personnel and human subjects blinded to study group during study?”. These studies recruited clinical populations (i.e., referred patients); therefore, blinding to study groups could not be achieved. Although these factors increase the risk of bias, refractive error is an objective measure of ocular biometrics, and the gfERG is an objective measure of retinal function, which together minimize the potential effect on internal validity.

### 3.3. Study Characteristics

Study characteristics are summarized in Table 6. The design of the included studies was either between group (37.50%), correlational (25%), within group (12.50%), or mixed (37.50%). A total of 522 participants were identified across all included studies, and where the information was provided, an approximately proportional number of female and male participants were included (37.50%). 

Participant age ranged from 7 to 50 years across all included studies. Fifty percent of studies reported age of participants in M(SD), ranging from 7.1(4.4) to 26.9(2.4) years. In the remaining 50% of the studies not reporting M(SD), age ranged from 10 to 50 years. Fifty percent of the studies assessed gfERGs in younger populations (10–23 years), while the remaining assessed older populations (7–50 years). One study did not report maximum age of the participants [43]. In total, 37.50% of studies reported that the controls were age-matched to the myopic/hyperopic participants. 

Myopia was assessed in 62.50% of the studies, with refractive state ranging from +0.5D to −27D, meanwhile refractive error ranged from 0 to +11D in the study assessing hyperopia (12.50%). Both myopia and hyperopia were assessed in 25% of studies, where refractive error ranged from ≤−6D to ≥+6D. Most participants were identified has having no pathologies secondary to myopia including retinal detachment, retinopathy, or any other ocular disease (62.50%). The remaining participants were predominantly characterized as either having some retinal degeneration (12.50%), reduced vision (12.50%), or posterior vitreous detachment (12.50%). 

Among the ERG stimulus types, 50% of studies reported employing International Society Clinical Electrophysiology of Vision (ISCEV) standards for photopic and scotopic flash in light and dark adapted conditions, respectively, while the remaining 50% of studies were published before ERG standard protocols for measurement were established [42,43,50,51]. These studies used customized photopic and scotopic stimuli for the ERG measurements. In 90% of the studies the participants were dark/light-adapted before ERG flash was delivered. International Society Clinical Electrophysiology of Vision protocols for ERG stimulus specifies ≥20 min for dark-adaptation and ≥10 min for light-adaptation [31]. Dark-adaptation duration across these studies ranged from 5 to 30 min. Light-adaptation duration across these studies ranged from 10 to 20 min. Participants were either dark-adapted (50%), both dark/light-adapted (37.50%), or neither (12.50%).

### 3.4. Effect of Refractive Errors on the gfERG Waveform 

ERG components frequently reported in the studies included a-wave amplitude (6/8 studies), b-wave amplitude (8/8 studies), a/b-wave ratio (7/8 studies), oscillatory potentials (OPs) (2/8 studies), and implicit time (6/8 studies), representing various aspects of cellular activity in the inner and outer retina [33,34]. However, for one study [44], it was not possible to extract quantitative data for wave amplitudes and implicit times, and therefore only qualitative data were provided.

### 3.5. Effect of Myopia on the gfERG

Results of individual studies assessing the effects of myopia are summarized in Table 7. A decrease in the photoreceptor-driven a-wave (3/5 studies) and bipolar-driven b-wave (5/6 studies) amplitude was the most frequent finding across these studies in both photopic and scotopic conditions. In one study (Sachidananda et al., 2017), scotopic amplitudes were more affected than photopic amplitudes. For 3/5 studies the a/b-wave ratio was unchanged in myopia, and, in 2/5 studies, the a/b-wave ratio was increased (particularly in individuals with high myopia). Implicit time of the major waves did not change between myopia and the comparison group in 5/5 studies reporting on this component. Of the studies reporting OP data, one study reported an increase in OP amplitude in scotopic conditions [40] while another reported the opposite effect in photopic conditions [41]. In the one study not reporting quantitative data for the changes observed, ERG results were indicated by proportions of abnormality detected in myopic patients. Abnormal a-wave to b-wave amplitude ratios were commonly reported in high myopes (≥−6D) [44]. 

### 3.6. Effect of Hyperopia on the ERG 

Results of individual studies assessing the effects of hyperopia are summarized in Table 8. Findings across studies assessing the effect of hyperopia on the ERG waveform were inconsistent. In the Perlman et al. [42] study, ERG results of 35% of the hyperopes were characterized by an increased a-wave and a decreased b-wave. Another group of hyperopes (19%) displayed increased b-wave amplitude, while the remaining (45%) displayed normal ERGs. Reassessment of a sub-group of these participants produced similar ERG profiles 8 years later in the Kennet et al. [50] study. No association between degree of refractive error and b-wave amplitude or a/b-wave ratio was found in this later study. In the Flitcroft et al. study [44], proportions of abnormal ERGs were increased in high hyperopes as in high myopes. 

## 4. Discussion

In the present systematic review, screening of 2099 records identified eight studies using gfERG to assess retinal functioning in myopes and hyperopes that met inclusion criteria. These studies demonstrated overall minimal risk of bias or otherwise justified risk of bias for some domains as assessed using OHAT guidelines. Changes in the major components of the ERG waveform including the a-wave amplitude, b-wave amplitude, and a/b-wave ratio were reported in both myopia and hyperopia. 

The most frequent finding from studies assessing myopia was b-wave amplitude attenuation. Although the exact source of the ERG b-wave remains disputed, it is typically accepted to primarily reflect the activity of mid-retinal ON-bipolar cells at light onset [33]. Therefore, this attenuation would suggest that signal transduction from the photoreceptors to the bipolar cells, or bipolar cell excitability at light onset, may be affected in the myopic eye. The a-wave amplitudes were similarly diminished, though this finding was less frequent. The a-wave reflects the activity of photoreceptors in outer retina [33], and hence reduction in its amplitude under photopic and scotopic conditions would suggest that the function of the photoreceptors is impacted in myopia. The a-wave to b-wave ratio provides an indication of whether signal transmission from the outer retinal photoreceptors to the inner retina is functioning normally [53,54], and this was typically unchanged between comparison groups. However, the reviewed studies identified b-wave attenuation more frequently than a-wave attenuation in myopia, and two studies reported an increased a/b ratio associated with high and pathological myopia [42,43], suggesting that transmission of the visual signal from photoreceptor to bipolar cells, or bipolar cell responses, may display proportionally more functional impairment as myopia progresses. 

Findings regarding implicit time and oscillatory potential components of the gfERG waveform were less frequently reported. Where information was available, implicit time between the onset of the response and maximum a-wave and b-wave amplitude did not differ between groups suggesting that the speed of signal transduction from the photoreceptors to the bipolar cells is not affected in myopia. Oscillatory potentials are generally thought to reflect the activity of amacrine and ganglion cells [35], though their exact origin remains unresolved. For studies reporting OP amplitudes, findings were mixed (with one study reporting an increase under scotopic conditions [40] and another a decrease under photopic conditions [41]). 

In contrast to myopia, only three studies assessing gERGs in hyperopic participants were identified. Inconsistencies in the reporting and findings of these studies hindered synthesis and interpretation of the results. Hyperopia is a far less common clinical condition in adults than myopia and is not increasing in worldwide prevalence [55,56], which may account for the lack of clinical gfERG studies of hyperopes.

The gfERG findings in human in human myopes reviewed here build on electrophysiological, structural, and pharmacological evidence from animal models implicating the photoreceptors and ON- and OFF- bipolar cell pathways in the control of eye growth and the development of myopia [18,19,20,57]. The human studies reviewed here consistently concur with the findings of gfERG studies in chick models of early and established myopia identifying decreased a-wave and b-wave amplitudes [36,37,38,39]. Using a quantitative model of phototransduction, Westbrook et al. [39] derived the photoreceptor light response in myopic and emmetropic chick eyes from the leading edge of the a-wave. This model demonstrated that photoreceptors in myopic eyes are significantly more sensitive to lower intensity light stimulus than normally developing eyes. However, at higher intensities, the photoreceptor light response declines faster in myopic than in control eyes, suggesting an increase in negative feedback mechanisms. This reduction in photoreceptor response to intense light is consistent with ultrastructural studies demonstrating that rod outer segments are elongated in both occlusion and lens defocus models of myopia [58,59,60]. Such a reduction in outer segment phagocytosis indicates that a level of photoreceptor inactivity may be involved in both growth paradigms (as disc shedding follows a circadian rhythm stimulated by dark–light transition).

Pharmacological and gene knockout studies in animals have provided further evidence for photoreceptor and bipolar cell involvement in ocular growth regulation. A number of early such studies examining the effects of neurotoxic substances in animal myopia models have indicated that agents which disrupt the majority of amacrine or ganglion cell functioning alone (while they may alter anterior chamber depth) have little effect on vitreous chamber growth [23,24,25,26]. In contrast, agents that also affect bipolar or photoreceptor functioning do alter the rate of postnatal vitreous enlargement and visually induced ocular growth [21,23,24,27,28], and pharmacological or genetic disruption of the balance between bipolar cell ON- and OFF- pathways has been shown to directionally alter ocular growth [20,21,36,57,61]. 

### 4.1. Limitations

Several limitations were common across the included studies. Not unexpectedly, some early studies did not follow ISCEV standards for testing, as they were conducted before the standards were established [42,43,50,51]. Therefore, methods of gfERG testing varied in these studies considerably. Studies that followed the ISCEV protocol for gfERG stimuli often failed to adhere to the durations outlined for dark-adaptation and light-adaptation. The current ISCEV standard specifies a minimum of 20 min for dark-adaptation and 10 min for light-adaptation. Failure to adhere to the duration of adaptation means the rod systems isolated by dark-adaptation and cone systems isolated by light-adaptation may have been inadequately assessed [31]. In some cases, reporting of the results did not provide clear indications about the direction of change for the outcomes measured. Reporting of the outcomes was too general in these instances to determine which aspects of the ERG waveform were affected and to what degree this effect was detected [42,44,50]. Furthermore, the included studies covered a wide age range, and few studies reported whether the comparison groups were age-matched [40,41,51]. Thus age matching remains a crucial factor to consider, given that age-related changes in the ERG have long been recognized [62,63], and the stability of refractive state and likelihood of secondary associated pathology vary across the life span [64].

### 4.2. Future Directions

The review identified that additional studies that include assessment of hyperopes are required to obtain a greater understanding of relative changes in function associated with signed-directional growth. In order to examine the involvement of the inner retinal contribution to the development of refractive errors, it would be beneficial for future reviews examine PERG waveform characteristics in myopia and hyperopia. Further, it is recommended that future investigations using gfERG should more consistently implement standardized testing protocols through the development of clinical guidelines for assessment and reporting gfERG to enhance the utility of this measure for examination of outer retinal changes associated ametropias in addition to ocular pathologies.

## 5. Conclusions

The global flash electroretinogram waveform appears to be altered in the myopic eye with reductions in the a- and b-waves the most frequently reported characteristics. The implicit times of global flash electroretinogram amplitudes remain unchanged between comparison groups, while a/b ratio increases have been associated with high myopia where secondary pathology may be present. While limited to global flash electroretinograms, the current review findings for impaired a-wave and b-wave activity suggest that the function of the rod and cone photoreceptor response to light onset is perturbed, and hence that transmission of the visual signal to the bipolar cells is likely to be reduced in human myopia, consistent with animal model studies. The effects of hyperopia on the global flash electroretinogram waveform are less conclusive given the limited number of studies and inconsistency in their reporting. Higher quality studies consistently and explicitly reporting on global flash electroretinogram outcomes are required to further clarify the evidence.

## Figures and Tables

**Table 1:** Database Search Strategy and Example Results from PubMed.

Search Terms	Search Results
1. myopi* OR short-sightedness OR near-sightedness OR hyperopi* OR long-sightedness OR far-sightedness OR emmetrop* OR “refractive error” OR “refractive adaptation” OR “refractive compensation” OR “refractive status” OR “refractive state”	37,353
2. “retinal functioning” OR “retinal neurophysiology” OR electroretinog* OR ERG OR electrophysiology	139,612
1 AND 2	754

**Table 2:** Hierarchy of Exclusion Reasons for Full-Text Screening.

Order	Reason for Exclusion
1	Full text not available (e.g., conference abstract)
2	Study not in English
3	Population does not fit inclusion criteria (e.g., non-human animals, or congenital stationary night blindness)
4	Wrong group comparison (i.e., does not compare ERG measures between different refractive error groups)
5	Intervention study with no baseline data
6	Wrong study design (e.g., case studies)
7	Wrong outcomes variables

**Table 3:** Data Extracted from Each Included Study for the Systematic Review.

Categories	Variables Extracted
Demographics	Sample size (*N*), sex (F, M), and age (M, SD, range)
Refractive error characteristics	Refractive error (M, SD, range) and presence/absence of secondary pathology
gfERG characteristics	Study design, stimulus (i.e., ISCEV photopic and scotopic flash), adaptation state (duration (mins) of dark-adaptation and light-adaptation), and changes in the amplitude and implicit time of all reported waves

**Table 4:** OHAT Risk of Bias Questions.

No.	Questions Assessing Risk of Bias
1	Was administration dose or exposure level adequately randomized?
2	Was allocation to study adequately concealed?
3	Did selection of study participants result in appropriate comparison groups?
4	Did the study design or analysis account for important confounding and modifying variables?
5	Were experimental conditions identical across study groups?
6	Were the research personnel and human subjects blinded to the study group during the study?
7	Were outcome data complete without attrition or exclusion from analysis?
8	Can we be confident in the exposure characterization?
9	Can we be confident in the outcome assessment?
10	Were all measured outcomes reported?
11	Were there no other potential threats to internal validity?

**Table 5:** OHAT risk of bias assessment of the included studies.

Citation	Q3	Q4	Q6	Q7	Q8	Q9	Q10
Blach et al., 1966 [43]	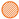	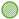	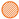	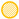	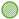	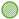	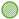
Flitcroft et al., 2005 [44]	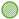	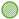	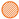	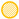	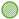	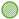	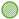
Kennet et al., 1993 [50]	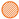	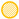	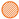	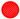	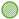	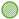	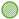
Perlman et al., 1984 [42]	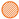	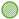	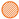	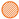	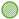	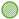	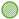
Sachidanandam et al., 2017 [52]	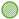	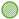	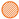	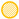	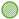	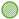	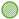
Wan et al., 2020 [40]	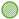	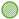	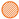	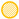	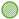	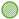	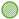
Westall et al., 2001 [41]	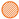	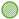	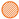	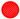	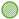	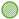	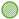
Yamamoto et al., 1997 [51]	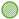	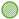	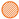	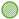	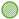	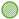	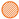

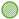
 definitely low 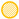 probably low 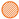 probably high 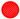 definitely high.

**Table 6:** Characteristics of Included Studies.

Study	Demographics		Refractive Error Characteristics		ERG Characteristics		
	Sample Sizes: *N* (Sex: M, F)	Age: *M* (*SD*), Range	Refractive Error: *M* (*SD*), Range	Secondary Pathology	Study Design	Stimulus	Adaptation State
Blach et al., 1966 [43]	My: 35 Em: 25	My: >10 y	My: −2 to −27 D	My: all show chorioretinal degeneration	Between groups	Photopic and scotopic square-wave flash	DA: 20 min
Flitcroft et al., 2005 [44]	Total: 123 (74 M, 49 F) High My: 15 Low My: 19 Em: 35 Low Hy: 44 High Hy: 10	7.1(4.4) y	High My: ≤−6D Low My: >−6D and ≤−0.75D Em: >−0.75 and <1.5D Low Hy: ≥1.5D and <6D High Hy: ≥6D	All patients had reduced vision of unknown cause	Between groups	ISCEV standard photopic and scotopic flash and 30 Hz flicker	DA: 5 min
Kennet et al., 1993 [50]	Hy: 25 (15 M, 10 F) Em: 10	15–23 y	Hy: 6.60(1.7)D, 5D to 11.5D Em: 0 to −2D	No retinal abnormalities	Within group and correlational	Photopic and scotopic flash	DA: 25 min
Perlman et al., 1984 [42]	High My: 7 Em: 26 Aphakia: 7 Hy: 31	High My: 15–50 y Aphakia: 15–50 y Hy: 7–25 y	High My: <−6D Em: Normal (not otherwise defined) Hy: >+5D	No retinal abnormalities except myopic crescents in eyes with high myopia	Correlational	Photopic and scotopic flash	DA: 25 min
Sachidanandam et al., 2017 [52]	Total: 100 (44 M, 56 F)	22.01(5.6) y	+0.5 to −18D	No pathology (including myopic retinopathy)	Correlational	ISCEV standard photopic and scotopic flash	LA: 20 min DA: 10 min
Wan et al., 2020 [40]	High My: 10 (4 M, 5 F) Moderate My: 11 (5 M, 6 F) Low My: 11 (4 M, 6 F) Em: 10 (4 M, 6 F)	High My: 26.0(2.2) y Moderate My: 26.9(2.4) y Low My: 25.3(2.5) y Em: 25.5(2.2) y	High My: −7.2(0.7), <−6.25D Moderate My: −4.5(0.8), −3.25 to −6D Low My: −2.4(0.6), >−3.00D Em: 0.1(0.1)	Participants with ophthalmological disease excluded	Between groups and correlational	ISCEV standard photopic and scotopic flash	LA: 20 min DA: 30 min
Westall et al., 2001 [41]	High My: 33 Low My: 8 Small RE (control): 19	High My: 31, 13–37 y Low My: 28, 24–37 y Small RE (control): 27, 20–36 y	High My: −8.78D, −6.00D to −14.50DLow My: −3.75D, −3.00D to −5.00D Small RE (control): −0.13D, +0.75D to −2.75D	All groups: No myopic retinopathy High my: *N =* 4 partial posterior vitreous detachment Low My: *N =* 2 lattice (no holes)	Between groups and correlational	ISCEV standard photopic and scotopic flash	LA: 10 min DA: 30 min
Yamamoto et al., 1997 [51]	High My: 12 Low My: 19 Em: 22	High My: 26.7(8.1) y Low My: 26.6(5.1) y Em: 25.5(4.6) y	High My: >−6.25D Low My: −3D to −6D Em: +2.5D to −2.5D (median −0.5D)	High my: No lattice or staphyloma	Between groups	Photopic chromatic flash ERGs to isolate cone responses	

Note. Sex: M, F = number of males and females, My = Myopia group, Hy = Hyperopia group, Em = Emmetropic controls, RE: Refractive error, y = years, D = Dioptres, ISCEV = International Society for Clinical Electrophysiology of Vision. DA = dark adaptation time, LA = Light adaptation time. Blank cells and partially complete cells are indicative of missing data.

**Table 7:** ERG Results of Studies Assessing Myopia.

Study	Amplitude				Implicit Time *	Note
	A-Wave	B-Wave	A/B-Ratio	OPs		
Blach et al., 1966 [43]	↑	↓	↑			a-wave amplitude increases were larger in early and moderate (relative to advanced) pathology groups. B-wave amplitude decreases were larger in myopes with advanced pathology.
Flitcroft et al., 2005 [44]	Abnormal in subset	Abnormal in subset	Abnormal in subset			An increased proportion of abnormal ERG results were obtained in patients with high ametropia (<−6D). The most common abnormalities for myopes were combined rod/cone defects and abnormal b-wave to a-wave amplitude ratios or abnormal on-off pathway responses. Quantitative data regarding wave amplitudes or implicit times were not reported.
Perlman et al., 1984 [42]	↓ (photopic and scotopic)	↓ (photopic and scotopic)	↑ (see note)		No change (b-wave photopic)	a/b ratio displayed no significant change in between-group comparisons but was inversely related to refraction in myopic patients.
Sachidanandam et al., 2017 [52]	↓ (photopic and scotopic)	↓ (photopic and scotopic)	No change		No change	Scotopic amplitudes were more affected than photopic amplitudes.
Wan et al., 2020 [40]	↑ (scotopic), No change (photopic)	↑ (scotopic), No change (photopic)	No change	↑ (scotopic), No change (photopic)	No change (photopic and scotopic a- and b-waves and OPs)	
Westall et al., 2001 [41]	↓ (scotopic and photopic)	↓ (scotopic and photopic)	No change	↓ (photopic)	No change	Amplitude decrease was greater for later (versus earlier) photopic OPs.
Yamamoto et al., 1997 [51]		↓ (s-cone ERG and L,M-cone ERG)			No change	

Note. * = Where available, implicit time findings for individual waveforms are reported; however, implicit times were commonly not reported or reports were incomplete or general. OPs = Oscillatory potentials. ↑↓ = indicates direction (i.e., increased or decreased) of change in the ERG component.

**Table 8:** ERG Results of Studies Assessing Hyperopia.

Study	Amplitude			Implicit Time *	Notes
	A-Wave	B-Wave	A/B-Ratio		
Flitcroft et al., 2005 [44]	Abnormal in subset	Abnormal in subset	Abnormal in subset		An increased proportion of abnormal ERG results were obtained in patients with high ametropia (>+6D). The most common abnormalities for hyperopes were combined rod/cone defects. Quantitative data regarding wave amplitudes or implicit times were not reported.
Kennet et al., 1993 [50]		No association with degree of RE	No association with degree of RE	No change (photopic b-wave)	Study reports reassessment of a subset of patients from Perlman et al. (1984). No significant changes in wave amplitudes were observed, suggesting that the subgroups identified 8 years earlier by Perlman et al. represented stable ERG profiles.
Perlman et al., 1984 [42]	↑ (36%), normal (45%),	↓ (36%), normal (45%), ↑ (19%)			Three subgroups were identified: Group 1 (*N* = 11) displayed increased a-wave and decreased b-wave amplitudes. Group 2 (*N* = 14) displayed normal ERG responses. Group 3 (*N* = 6) displayed increased b-wave amplitudes.

Note * = Where available, implicit time findings for individual waveforms were reported; however, implicit times were commonly not reported, or reports were incomplete or general. Oscillatory Potentials (OPs) were not reported, and thus this column was excluded from the table. RE = Refractive error. ↑↓ = indicates direction (i.e., increased or decreased) of change in the ERG component.

## Data Availability

The data extracted for this paper are available in the original research articles that were systematically reviewed.
